# Mesenchymal Stem Cells: Current Concepts in the Management of Inflammation in Osteoarthritis

**DOI:** 10.3390/biomedicines9070785

**Published:** 2021-07-07

**Authors:** Asma Abdullah Nurul, Maryam Azlan, Muhammad Rajaei Ahmad Mohd Zain, Alphy Alphonsa Sebastian, Ying Zhen Fan, Mh Busra Fauzi

**Affiliations:** 1 School of Health Sciences, Universiti Sains Malaysia, Kubang Kerian 16150, Kelantan, Malaysia; maryamazlan@usm.my (M.A.); alphyalphonsa1@gmail.com (A.A.S.); yingzhen2008@hotmail.com (Y.Z.F.); 2 Department of Orthopaedics, School of Medical Sciences, Universiti Sains Malaysia, Kubang Kerian 16150, Kelantan, Malaysia; rajaei@usm.my; 3 Centre for Tissue Engineering and Regenerative Medicine, Faculty of Medicine, Universiti Kebangsaan Malaysia, Cheras, Kuala Lumpur 56000, Malaysia; fauzibusra@ukm.edu.my

**Keywords:** cartilage regeneration, extracellular vesicles, inflammation, mesenchymal stem cell, osteoarthritis

## Abstract

Osteoarthritis (OA) has traditionally been known as a “wear and tear” disease, which is mainly characterized by the degradation of articular cartilage and changes in the subchondral bone. Despite the fact that OA is often thought of as a degenerative disease, the catabolic products of the cartilage matrix often promote inflammation by activating immune cells. Current OA treatment focuses on symptomatic treatment, with a primary focus on pain management, which does not promote cartilage regeneration or attenuate joint inflammation. Since articular cartilage have no ability to regenerate, thus regeneration of the tissue is one of the key targets of modern treatments for OA. Cell-based therapies are among the new therapeutic strategies for OA. Mesenchymal stem cells (MSCs) have been extensively researched as potential therapeutic agents in cell-based therapy of OA due to their ability to differentiate into chondrocytes and their immunomodulatory properties that can facilitate cartilage repair and regeneration. In this review, we emphasized current knowledge and future perspectives on the use of MSCs by targeting their regeneration potential and immunomodulatory effects in the treatment of OA.

## 1. Introduction

Osteoarthritis (OA) is the most common form of degenerative joint disease, typified by degeneration of cartilage and osseous overgrowth. The incidence of OA occurs in OA affects 13.9% of individuals aged 25 and older, and 33.6% of those over the age of 65 [1]. According to the Arthritis Foundation, more than 30 million people in the United States have OA, with the cases of knee OA are the most common [2]. The most common features of OA include pain, tenderness, swelling, stiffness and locking around the affected joint [3]. The pathogenesis of OA dictates the predominance of destructive processes by altering the tissue homeostasis of articular cartilage and subchondral bone.

OA is characterized by the breakdown of joint cartilage and subchondral bones. Articular cartilage is an essential structural component of the human body. It is composed of specialized cells known as chondrocytes. These chondrocytes generate a substantial number of extracellular matrix (ECM), which is made up of collagen fibers, elastin fibers and proteoglycan. Under physiological conditions, this matrix undergoes a constant remodeling process where both degradative and synthetic enzymes known as matrix metalloproteinases (MMPs) are tightly regulated [4]. These MMPs are released as inactive proenzymes that require enzymatic cleavage to become activated. Once active, MMPs are susceptible to the plasma-derived MMP inhibitor, alpha-2-macroglobulin, as well as tissue inhibitors of MMPs (TIMPs), which are also produced by synovial cells and chondrocytes.

In OA, the MMPs such as collagenases, stromelysins and gelatinases are overexpressed, changing the balance in favor of net degradation and resulting in the loss of collagen and proteoglycan from the matrix [5]. Subsequently, chondrocytes will proliferate and produced increased amounts of proteoglycan and collagen molecules in response to the loss signals. The presence of inflammatory cytokines, interleukin-1β (IL-1β) and tumor-necrosis factor-α (TNF-α), drive the catabolic pathways and perpetuate the progression of OA [6]. However, as the disease progresses, reparative measures are overwhelmed by progressive cartilage breakdown. These lead to the disease progression where the cartilage becomes softer, pitted and rough, and gradually disintegrate from the bone ends. The linings of joints might become inflamed and thickened. In addition, muscles around the arthritic joint become weaker and nerves become more sensitive. These changes might limit movement and result in pain.

Current OA therapeutic approaches focus on pain relief and symptom control rather than treating or slowing progression of the disease. Hence, there is an urgent need to explore new therapeutic strategies for OA. Cell-based therapies which involve the delivery of mesenchymal stem cells (MSCs) to the osteoarthritic joint have emerged as a promising strategy to the current treatment by targeting their regeneration potential and immunomodulatory effects. In this review, we discuss the current status of stem cell therapies, and also recent advancements and future perspectives in MSC therapy for OA.

## 2. Inflammation in Osteoarthritis

**Figure 1:**
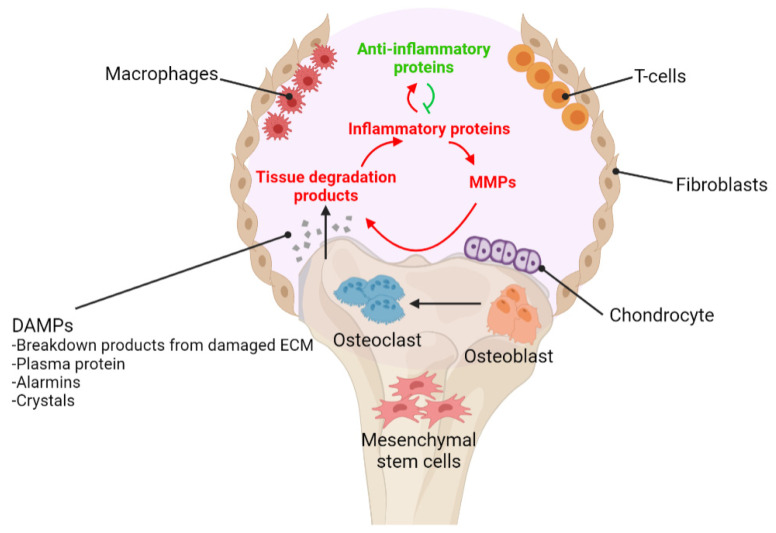
**Schematic representation of inflammation process in osteoarthritis**. OA is defined by the progressive breakdown of articular cartilage and subchondral bone, and also low-grade inflammation. Following joint injury, cartilage tissue damage causes the production of damage-associated molecular patterns (DAMPs), which include cartilage extracellular matrix (ECM) breakdown products and intracellular alarmins that signal pattern recognition receptors on synovial macrophages, fibroblasts, T cells, or chondrocytes to induce the local release of inflammatory mediators. These activated cells will produce inflammatory factors such as cytokines, chemokines, and catabolic enzymes, either directly or indirectly by inducing proteolytic enzymes which will accelerate cartilage destruction in the progressing osteoarthritis.

OA is often regarded as a degenerative disease in contrast to rheumatoid arthritis; however, recently the degenerative theory is no longer acceptable due to increasing evidence suggesting that inflammation also plays important role in the initiation and aggravation of OA. An early observation by Homandberg and colleagues [7] suggested that ECM breakdown products enhanced inflammation and cartilage breakdown, as well as pro-inflammatory mediators (Figure 1). Their observations supported a theory in which damage resulting in ECM breakdown produces damage associated molecular patterns (DAMPs) capable of triggering local inflammatory responses, resulting in increased chondrolysis and the release of more ECM breakdown products. There are numerous ECM breakdown products that have been recognized as DAMPs mediate cartilage damage which include fibronection [8], biglycan [9], tenascin C [10] and hyaluronic acid [11].

Several animal and human clinical studies have shown that chondrocytes, synovium and other surrounding tissues can release pro-inflammatory mediators even in the absence of overt inflammation, and various pathways culminate on aggrecanases and collagenases activation in OA [12]. In OA, not only chondrocytes, but also the cells in the synovium and other joint tissues, become activated due to exposure to aberrant environmental factors such as high-magnitude mechanical stress, inflammatory cytokines as well as ECM proteins [13]. The activation of stress-and inflammation-induced signaling, transcriptional and post-transcriptional activities can result in phenotypic shifts, apoptosis and abnormal expression of inflammation-related genes, including catabolic genes [14]. These include nitric oxide synthase (NOS)-2, cyclooxygenase (COX)-2 and several MMPs, including MMP-13, and a disintegrin and metalloproteinase (ADAM) with thrombospondin-1 domains (ADAMTS)-4 and 5.

Following cartilage damage, the immune system is activated in the joint lining which further triggers synovitis. However, as OA progresses, inflammation can occur surround the damaged joint. This process triggers both innate and adaptive immune systems activation exemplified by the presence of activated macrophages, elevated production of proinflammatory cytokines, activation of complements as well as toll-like receptors (TLRs) which play significant roles in the progression of the disease [15]. Previous study has demonstrated that fibronectin fragments stimulated the release of proinflammatory cytokines, including TNF-α and IL-1β, as well as MMP1 and MMP3 [7]. 

Cytokines that have been linked to the pathogenesis of OA include TNF-α, IL-1, IL-2, IL-6, IL-15, IL-17 and IL-21 and several chemokines [16]. Among these cytokines, TNF-α and IL-1β are the main cytokines involved in the pathogenesis of OA. IL-1β promotes catabolic effect and inflammatory reactions in the articular cartilage thus leads to the destruction of the cartilage. It was previously reported that patients with OA have an increased level of IL-1β and TNF-α in the synovial fluid, cartilage, synovial membrane and the subchondral bone layer [17]. TNF-α and IL-1β are also responsible in the progression of OA by promoting the release of proinflammatory cytokines such as IL-6 [18], IL-17A [19] and chemokines such as IL-8 (CXXL8), vascular endothelial growth factor (VEGF) [19] and CC-chemokine ligand 5 (RANTES) [20]. Osteoarthritic chondrocytes treated with IL-1β demonstrated catabolic responses by promoting degeneration of cartilage through upregulation of MMPs [7]. In addition, TNF-α and IL-1β also promote the production of ADAMTS metalloproteinases by the chondrocytes, which are responsible for the proteolysis of aggrecan molecules [21]. 

IL-1β and TNF-α are also involved in the stimulation of various inflammatory mediators implicated in OA. For instance, chondrocytes treated with TNF-α and IL-1β upregulated the expression of genes encoding inducible nitric oxide synthase (iNOS), COX-2 and microsomal prostaglandin E synthase 1, soluble phospholipase A2, and also stimulated the release of nitric oxide (NO) and prostaglandin E_2_ (PGE_2_) [22]. NO and PGE2 stimulate MMP activation and production, inhibit the synthesis of anabolic macromolecules such as collagen and proteoglycan, inhibit the generation of IL-1Ra, and promote chondrocyte apoptosis, all of which contribute to joint inflammation and degradation [23]. These cytokines also stimulate the formation of reactive oxygen species (ROS), primarily NO and the superoxide anion, which produce hydrogen peroxide, peroxynitrite, and hydroxyl radicals, which cause cartilage degradation [24]. Furthermore, IL-1β and TNF-α suppress the production of antioxidant enzymes that scavenge ROS, such as superoxide dismutase, catalase, and glutathione peroxidase, exacerbating the detrimental effects of ROS on cartilage [25].

Similar to IL-1β and TNFα, IL-6 also regulates the catabolic responses by promoting degeneration of cartilage via the production of MMPs as well as alters the production of type II collagen [26]. IL-6 has an enormous effect on bone, by regulating the bone resorption through the osteoclast activity. During OA progression, increased production of IL-6 promotes osteoclastogenesis thus causes changes in the subchondral bone layer [27]. Limited studies described the involvement of IL-17A in OA. Askari [28] suggested the regulatory role of IL-17A in the pathogenesis of OA as indicated by significant serum level of IL-17A in OA patients compared to normal subjects [28]. Another study found IL-17 in the synovial fluid of patients with end-stage knee and hip OA, indicating that IL-17 is involved in the pathogenesis of OA [29].

The involvement of TLRs in the pathogenesis of OA has also been reported. A previous study has shown that ECM-derived DAMPS or alarmins act as ligands for TLRs that may lead to the activation of catabolic events and downstream inflammatory responses in articular cartilage [30]. In OA animal model, the level of TLR-2 and TLR-4 were elevated in the cartilage of the lesion areas following activation by the ligands, peptidoglycan, and lipopolysaccharide [11]. Increased activation of these TLRs leads to increased expression of downstream inflammatory and catabolic genes, such as MMP-3, MMP-13 and NOS2, via the cytosolic adaptor MyD88 and subsequent NF-κB signaling [11]. However, a study in knock-out-TLR 1, 2, 4, 6 and MyD88 of OA mouse model with partial meniscectomy showed no effect on OA severity [31].

## 3. Etiology

The etiology of OA is complex and to date is not fully understood. However, there are few factors that may lead to the occurrence of OA. Age is considered the major independent risk factor of OA. The mechanisms by which age impacts cartilage health are diverse and complex, but they are most likely not just the result of cumulative “wear and tear” over time. As aging progresses, there are changes that occur not only in the cartilage, but also in other joint tissues including synovium, subchondral bone and muscle [32]. Thus, it is becoming evident that ageing changes in the musculoskeletal system, in concert with other factors, both intrinsic (e.g., alignment, over-loading) and extrinsic (e.g., genetics) to the joint, contribute to the development of OA. [33].

Osteoarthritis of the knee is found to be more prevalence in women than in men due to many factors including hormonal influences on cartilage metabolism, gender variation in risk of injury and the differences in the biomechanical activities of the knee [34]. However, the finding showed no gender differences in hip and hand OA. Obesity is another factor that is commonly recognized as a risk factor for the onset and progression of OA. Obese individuals are highly prone to knee OA due to mechanical load on weight-bearing joints [35] and also low-grade systemic inflammation through adipokines [36]. Obese people had considerably more severe joint degeneration in the knees than normal weight or underweight people, indicating that body weight impacts the severity of OA [37]. Two prospective cohort studies indicated that obesity is highly linked to an increased risk of knee replacement for OA [38]. A recent study showed a dose–response relationship between BMI and the clinical consequences of knee OA. The study demonstrated the pain score, WOMAC function score, and physical activity levels differed significantly among different BMI groups [39]. Moreover, a previous study has linked mechanical stress to inflammation in OA due to the presence of chondrocyte surface mechanoreceptors [40]. Previous studies have shown that mechanical stretch enhanced the expression of IL-1β and COX-2 and the amount of PGE_2_ synthesis [41] and MMP-2 expression in fibroblast-like synoviocytes [42]. In pre-clinical studies, the progression of OA occurs in mouse obesity model induced with high-fat diet caused elevation of IL-1β, IL-6, IL-8 and TNF-α [43]. These cytokines further stimulate the NF-κB signaling to trigger the articular chondrocyte catabolic process through the upregulation of matrix metalloproteinases (MMPs) [16]. 

Sports injury is common in active or young adults and knee injury enhances the risk for OA. As the year progresses 41-51% of participants with a history of knee injury develops radiographic signs of knee OA [44]. Most common sport injuries such as tear in cartilage tissue and ligament and joint dislocation may lead to development of OA [45]. Previous studies have reported that the chronic low-grade inflammation present in OA leads to disease progression. In medical conditions such as in Diabetes mellitus, elevated level of IL-1β and TNF-α leads to activation of NF-κB signaling in cells lining the synovial cavity and chondrocytes [46]. Genetic predisposition is also relevant in OA. For instance, alteration in TGF-β, Wnt/β-catenin, Indian Hedgehog, Notch and fibroblast growth factor (FGF) pathways are involved in the progression and development of OA [47]. The contribution of genetics in OA is estimated to be between 40% and 80%. It was found that a stronger genetic contribution occurs in hip OA than knee OA [48].

## 4. Current Therapeutics for OA

The Osteoarthritis Research Society International (OARSI), the American College of Rheumatology, and the American Academy of Orthopaedic Surgeons (AAOS) have outlined three therapeutic approaches for OA: physical measures, pharmacological therapy and surgery [49].

### 4.1. Physical Therapy

Physical therapy is related to daily activities. Living a healthier life with a balanced food and physical activities could be an adjunct therapy for OA. Overweight or obese people are at risk should benefit from weight loss as it can reduce the mechanical stress, lessen joint pain, hence reduce OA risks [50]. Studies have reported that moderate exercise can help strengthen muscles and delay the progression of OA [51]. Furthermore, exercise therapy is particularly helpful in decreasing pain and improving joint mobility as reported previously [52,53].

### 4.2. Drug Therapy

Pharmacologic treatment in OA is mainly aimed to alleviate pain. According to OARSI and AAOS, acetaminophen is considered as the first-line drug therapy in mild-to-moderate OA [54]. As shown in a Cochrane meta-analysis, taking acetaminophen reduced pain by four points (on a 0 to 100 scale) compared to placebo and resulted in a 5% improvement from baseline [55]. However, acetaminophen did not show significant immediate effect in improving function and showed short-term improvement of function [56]. 

Nonsteroidal anti-inflammatory drugs (NSAIDs) have long been used to treat moderate-to-severe OA because they provide anti-inflammatory and analgesic properties. NSAIDs function by inhibiting cyclooxygenase-2 (COX-2) and COX-1 enzymes, both of which are involved in prostaglandin synthesis. There are several classes of NSAIDs which include acetylsalicylic acid (aspirin), ibuprofen, naproxen and celecoxib. Eight randomized controlled trials have reported that NSAIDs were superior to acetaminophen in terms of pain alleviation [57]. In a systematic review, the efficacy and safety of acetaminophen and NSAIDs (ibuprofen, diclofenac, arthrotec, celecoxib, naproxen, rofecoxib) for the treatment of knee OA were evaluated [55]. Several studies have also suggested that NSAIDs were better than acetaminophen for improving moderate-to-severe pain in people with knee OA. However, the usage of NSAIDS was strictly restricted due to the occurrence of adverse effects in certain people (~30%) [58].

Opioids such as oxycodone, morphine or atypical opioid tramadol were used for the management of moderate-to-severe pain when NSAIDs and acetaminophen were ineffective or contraindicated [59]. Between 2003 and 2009, there was an increase in opioid use among OA patients, with 40% of knee OA patients receiving opioids in 2009 [60], while a more recent research from 2007 to 2014 showed a decrease to 15.9%, with prescription rates remaining relatively steady [61]. The use of opioids has been associated with various adverse effects, including nausea, dizziness, vomiting, constipation, tiredness, sleepiness and headache which lead to restriction to usage despite of their benefits in pain relief [61,62].

### 4.3. Intra-Articular Injections

Intra-articular injections of glucocorticoids and hyaluronic acid is recommended by OARSI as an alternative treatment for knee and hip OA [59]. The intra-articular injection is mainly indicated for the management of patients with moderate-to-severe pain who poorly responded to oral analgesic and anti-inflammatory drugs. Glucocorticoids administration is normally aimed to alleviate pain and reduce inflammation [63]; however, when taken in high doses over longer period of time will lead to detrimental effects [64]. While hyaluronic acid supplementation functions to alleviate symptoms in OA joint [65] by improving the synovial fluid function [66]. The efficacy and safety profiles of intra-articular glucocorticoids and hyaluronic acid are considered comparable [67]. Intra-articular injection of platelet rich plasma (PRP) is considered as a feasible and potential treatment of OA, as it contains different types of tissue growth factors including transforming growth factor β (TGF- β), platelet-derived growth factor, insulin-like growth factor-1 (IGF-1) and hepatocyte growth factor which have functions to promote chondrogenesis, increase angiogenesis and epithelial cell, osteoblast and fibroblast proliferation [68,69,70] as well as promote the production of hyaluronic acid and collagen [71]. Wang-Saegusa et al. [42] demonstrated clinical improvement in OA treatment with no adverse effects after introducing 3 intra-articular injections of autologous plasma PRP. Gormeli et al. have suggested multiple injections of PRP for better clinical outcomes in early OA patients [72]. However, there is a concern on the heterogeneity and lack of standardization in PRP preparation leading to difficulty to identify the exact content that was being injected into the affected knee [73].

### 4.4. New OA Drugs

Demands for new drugs for the management of OA are high due to inefficiency and multiple side effects from the current treatments. New drugs are different based upon their therapeutic targets include chondrogenesis inducers, matrix degradation inhibitors, apoptosis inhibitors, osteogenesis inhibitors and anti-inflammatory cytokines [74]. For example, recombinant human BMP-7, also known as osteogenic protein-1 (OP-1), has been introduced as the treatment for symptomatic knee OA [75]. The four different classes of BMP-7 were injected intra-articularly into OA patients averaging 60 years old, with treatments including 0.1 and 0.3 mg of BMP-7 demonstrating better improvement in symptoms. BMP-7 was predominantly used for the management of bone non-unions and spine fusion [76]. Two randomized placebo-controlled studies were conducted using interleukin (IL)-1β inhibitor demonstrated that the treatment did not significantly improved the OA symptoms compared to placebo [77,78]. 

### 4.5. Surgery

Surgery is recommended when both the pharmacology and non-pharmacology treatments for OA fail to reduce pain and restore functions. Total joint replacement is also recommended where the damaged joint is replaced with an artificial one [79]. Total joint replacement is more common to replace knee or hip damage, but it is also performed for hand joint such as carpometacarpal and interphalangeal joints [80]. However, an artificial knee does not function like the original joint in that it does not permit natural rotation or bending of the knee [81]. Another surgical procedure that can be carried out as a treatment option for OA is microfracture. Microfracture is a marrow stimulation method that permits contact between the joint space and subchondral bone marrow in order to release mesenchymal stromal/stem cells (MSCs) from the marrow and generate repair tissue. However, the repair tissue with microfracture usually degenerates over time especially in fractures bigger than 2 cm^2^, resulting in the development of scar-like fibrous tissue or even replacement with bone [82].

## 5. Cell-Based Therapy

The cell-based therapy has been initiated for cartilage repair since 1980s. A technique known as autologous chondrocyte implantation (ACI) was first described by Brittberg and colleagues in 1994 [83]. This technique used a combination of surgical and cell culture methods that require two stages of procedures [84]. Firstly, cartilage biopsy was obtained from the healthy area of the articular cartilage in patient, followed by chondrocytes isolation from the cartilage tissue using collagenase. The chondrocytes were then grown in a monolayer culture before being transplanted over the cartilage defect in the second stage procedure, either in suspension beneath a periosteal flap or synthetic membranes, or in three-dimensional matrices [85].

Over the past two decades, three generations of ACI have been developed based on the different types of implantation procedures. In the first generation, the periosteum retrieved from the patient’s tibia were sutured over the defect. The cell suspension was then introduced to the chamber. The disadvantage for the first generation is periosteal delamination. However, in the second generation, it is purely based upon bilayer collagen membrane. This is also sutured over the defect similar to the first generation to form a chamber where the chondrocytes are introduced. More interestingly, in the third generation, cultured chondrocytes are pre-seeded on a 3D-scaffold and then trimmed to fit the size of the defect. Later, it is implanted into the defect area with fibrin glue. Therefore, third generation ACI is called matrix-induced autologous chondrocyte implantation (MACI). The advantages of MACI over conventional ACI is that it reduces the surgical time and the fixation invasion, this in turn gives rise to a long-term cell maintenance.

Success stories of ACI have been reported widely. Brittberg [83] has reported symptomatic relief and eliminated knee locking in 14 out of 16 patients at 2 years follow-up after ACI. In a randomized controlled trial of 118 patients who had ACI or microfracture to treat cartilage defects in the knee, they discovered that both treatments provided comparable clinical results, but ACI was superior due to improved structural healing [86]. A recent study [85] found that after 20 years of follow-up from 23 patients (24 knees) who received the first-generation of ACI, 15 out of 24 knees showed significant improvement in all clinical parameters except stiffness, and the patients were able to retain their native knees with satisfactory results.

Despite the promising long-term clinical results, there are several limitations to the use of ACI. One of the major limitations is the biological response of the periosteal flap that causes detachment, delamination and late periosteal hypertrophy [83]. However, by replacing the periosteal flap with collagen sheets or resorbable scaffolds, adverse events and the aforementioned side effects in ACI have been effectively reduced [87]. Another limitation related to ACI is the structural variability which is caused by disorganized fibrocartilage [88]. The length of rehabilitation after ACI procedure can be a major limitation as well in this treatment option in order to allow the repaired tissues to have adequate time to remodel and mature. This can contribute to the delay of return to sports as long as 18 months [89]. Furthermore, chondrocytes are difficult to grow in a monolayer culture because they dedifferentiate into a fibroblast-like phenotype and lose their ability to produce stable cartilage, which may lead to structural variability [90,91]. The instability of the chondrocyte phenotype in the in vitro culture has encouraged more research on identifying alternative cells for cartilage tissue engineering. The most promising cell source that are commonly used in cartilage tissue engineering is mesenchymal stem cells.

## 6. Mesenchymal Stem Cells for Cartilage Tissue Engineering

**Figure 2:**
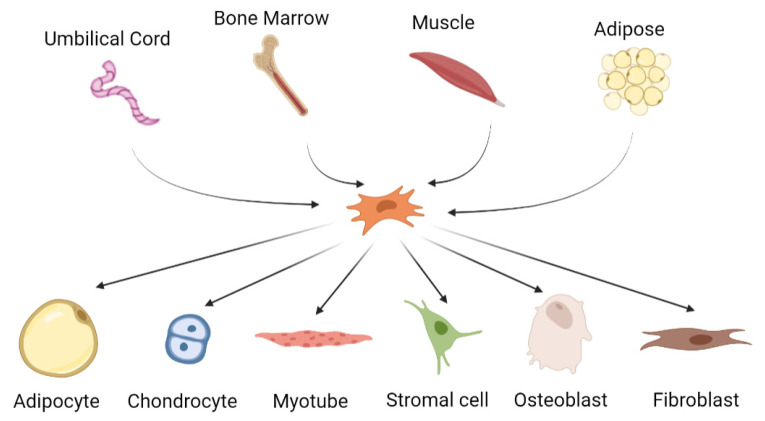
**The origin and differentiation potential of mesenchymal stem cells**.

MSCs are multipotent cells that can be differentiated into several types of cells including chondrocytes, adipocytes, osteoblasts and myogenic and neuronal cells [92,93,94,95,96] (Figure 2). MSCs can be isolated from various sources, primarily bone marrow, adipose tissue, dental pulp, placenta and umbilical cord, as well as from the skeletal tissues. They are characterized by their fibroblastic shape and specific marker expressions such as CD11b^+^, CD14^−^, CD34^−^, CD45^−^, HLA-DR^−^, CD73^+^, CD90^+^ and CD105^+^ [97].

Isolation of MSCs was first reported by Friedenstein and co-workers through their early works in the 1960s and 1970s [98,99]. The first isolated MSCs were identified in bone marrow, where the cells showed osteogenic potential and distinguished themselves from the majority of hematopoietic cells by their rapid adherence to tissue culture vessels and fibroblast-like appearance of their progeny in culture. In addition, in vivo transplantation of the cells led to differentiation of the cells into multiple skeletal tissues (bone, cartilage, adipose and fibrous tissues) confirming the multipotential of the cells [100]. Following the report by Friedenstein and colleagues, Owen and Caplan had demonstrated the presence of non-hematopoietic adult stem cell in the bone marrow in the late 1980s [101,102]. Later, the term mesenchymal stem cell was introduced by Caplan in 1991 [102] through their success in isolating human bone marrow-derived MSCs.

Unlike chondrocyte cells, MSCs are easy to expand in culture and exhibits chondrogenic potential. The use of MSC-differentiated chondrocytes is a promising strategy for cartilage regeneration. Chondrogenic differentiation from MSCs is well documented from various sources and techniques. Differentiated cells display the important cartilage-specific markers such as collagen type II, aggrecan and sulphated proteoglycans [103]. Similar to other tissue engineering approach, chondrogenic differentiation could be achieved in the presence of inducers, in this case the most established inducer is TGF-β [103], although other inducers such as bone morphogenetic proteins (BMPs) [104] and IGF [105] were also reported. MacKay et al. [106] used a combination of 100 nM dexamethasone and 10 ng/mL TGF-β3 and successfully induced chondrogenic differentiation of MSCs characterized by ECM with collagen type II, aggrecan and proteoglycan. Enhanced chondrogenic potential of bone marrow MSCs in a presence of combination treatments TGF-β3/BMP-6 and TGF-β3/IGF-1 were also reported [105]. The importance of TGF-β signaling in chondrogenic development was confirmed in a study which showed adipose tissue-derived MSCs that did not express TGF-receptor-1 protein had a lower chondrogenic capacity [107]. Progress in the chondrogenic differentiation potential of MSCs has led to the advancement of cultivation of the cells. MSCs co-cultured with juvenile articular chondrocytes (ACs) [108] with a presence of TGF-β3 in lower concentration [109,110] resulted in efficient chondrogenic differentiation.

Given their highly chondrogenic potential in in vitro culture, MSC-based therapy is among the promising therapeutic approaches to treat OA. Pre-clinical studies demonstrated encouraging data on therapeutic potential of MSCs in OA animal models. Bone marrow MSCs were implanted onto osteochondral defect which artificially made on 16 rabbits. MSCs-implanted rabbits demonstrated improved histological scores as well as enhanced production of collagen type II in the matrix [111]. Other pre-clinical studies showed the success of intra-articular injections of MSCs in goat [112] and porcine [113] models with improved cartilage healing of chondral defects.

In 2002, Wakitani and colleagues transplanted bone marrow MSCs into the articular cartilage defects in knees of 12 patients [114]. Even though there was no significant clinical improvement after six months, arthroscopic and histological grading scores were better than the control group. This study somehow highlighted the availability of autologous MSC culture thus sparked for more research using the cells. Currently, 74 clinical studies in various phases involving MSCs for OA are referenced at ClinicalTrials.gov. Centeno and colleagues [115] described percutaneous injection of bone marrow MSC which resulted in significant cartilage growth, decreased pain and increased joint mobility in the patient. Later, they published a case study of 339 patients, showing that of those patients who needed total knee replacement surgery (69% of the patient group), only 6.9% required replacement surgery again following MSC treatment. The study reported that 60% of patients showed >50% pain relief, while 40% reported >75% pain relief at 11 months [116]. In a randomized controlled trial, 30 patients with persistent knee pain who had not responded to conservative therapies improved in several functional indices and cartilage quality after intra-articular injections of bone marrow MSCs [117]. In a Phase II clinical trial of allogeneic MSCs, the safety and efficacy of intra-articular injection of Stempeucel^®^ in 60 patients with OA of knee were determined [118]. This study found that allogeneic transplant of Stempeucel^®^ was safe, with improved outcome in pain management scores in the lowest dosage (25 million cells). Regardless of its safety and efficacy, intra-articular injection of Stempeucel^®^ especially in the highest dosage (150 million cells) exhibited some adverse effects in the patients, but the adverse effects completely recovered upon symptomatic treatment.

Another source of MSCs that is also utilized for OA treatment is adipose-derived MSCs (ADSCs). As reviewed by Hurley [119], there were 16 studies that reported the use of ADSCs for the treatment of OA with various approaches. ADSCs harvested from infrapatellar fat pad prepared in platelet-rich plasma (PRP) injected into the OA knee showed improved mobility and function and reduced pain scores with no adverse effects [120]. At two years follow-up, patients had significantly improved pain scores as well as cartilage regeneration as confirmed by MRI [121]. In a study by Bui et al., non-expanded stromal vascular fraction (SVF) isolated from the adipose tissue and prepared in PRP had been delivered into 21 patients with grade II and III OA and reported significant improvements in pain score as well as increased thickness of the cartilage layer [122]. Similar procedure and outcome had been described by Bansal et al., which reported reduction in pain levels after 3 months injection with SVF prepared in PRP [123]. More importantly, autologous ADSCs transplantation reported in these studies offered minimal risk of side effects without graft rejection or tumorigenesis in the recipients, thus provide promising approach for OA treatment.

MSC-derived extracellular vesicles (EVs) are a diverse population of heterogeneous membranous vesicles and enriched in many bioactive molecules such as lipids, proteins, mRNAs, transfer RNA (tRNA), long non-coding RNAs (lncRNAs), microRNAs (miRNAs) and mitochondrial DNA (mtDNA) [124]. These molecules establish an EVs-mediated transport system which important in intercellular communication to regulate a wide range of physiological and pathological processes and pathways [125]. MSC-derived EVs have been widely documented to play important roles in the regulation of numerous cell activities such as cell proliferation, differentiation, migration and extracellular matrix synthesis [126,127,128]. When MSCs produce EVs, they encapsulate nucleic acids, proteins, and lipids from donor cells and transfer them to recipient cells such as resting stem cells in the stem cell niche or injured cells in the traumatic microenvironment [129,130]. These EVs-cell communications will stimulate regeneration by activating resting stem cells or restoring the functionality of the injured cells. EVs that have been shed by MSCs exhibit similar properties such as functional tissue repair and regeneration as their cells of origin and some studies reported added beneficial effects of MSC-derived EVs [131,132]. Moreover, several reports have demonstrated that MSC-derived EVs showed promising findings on cartilage repair and regeneration by regulating immunomodulatory activity, promoting regenerative capacities, diminishing apoptosis, and increasing proliferation [54,74,133,134].

Exosome derived from human embryonic stem cell-induced MSCs (ESC-MSCs) exhibited remarkable cartilage regeneration in osteochondral defects rat which characterized by complete restoration of hyaline cartilage [74]. Another study reported by Wang et al. also demonstrated that exosome derived from ESC-MSCs alleviated cartilage destruction and matrix degradation in the destabilization of the medial meniscus (DMM) model by increasing collagen type II (ColII) and aggrecan expressions but reducing ADMTS5 expression as well as improved the maximal and total OARSI scores which resulted in milder OA pathology [135]. Similar findings were demonstrated by Cosenza et al. in their in vitro and in vivo models [136]. Exosomes and microparticles isolated from BM-MSC increased anabolic cartilage markers (collagen type II, aggrecan) expression in OA-like chondrocytes in a dose-dependent manner, inhibited catabolic (MMP-13, ADAMTS5) and inflammatory (iNOS) markers, and decreased articular cartilage damage and subchondral bone degradation. Furthermore, EVs isolated from human adipose-derived MSC increased the proliferation and migration of human OA chondrocytes in vitro and decreased the progression of OA and protected cartilage from degeneration in both the monosodium iodoacetate (MIA) rat and the surgical destabilization of the medial meniscus (DMM) mouse models [133]. Another recent study found that exosomes derived from 3D culture of umbilical MSCs had better chondroprotective effects than exosomes derived from 2D culture systems, significantly stimulating chondrocyte proliferation, migration, and matrix synthesis, as well as improving gross appearance and attenuating cartilage defect in the animal model [137]. To date, no clinical trial has been conducted using MSC-derived EVs on osteoarthritis.

## 7. MSCs for the Management of Inflammation in OA

**Figure 3:**
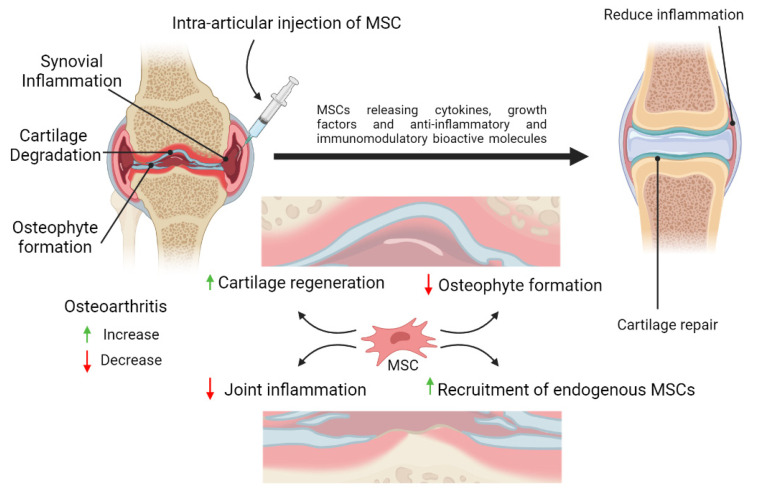
**Schematic representation of the potential therapeutic strategies utilizing mesenchymal stem cells (MSCs) therapy for cartilage repair and regeneration.** MSCs possess anti-inflammatory and immunomodulatory properties which could reduce inflammation in the joint. MSCs may also assist in the healing process by differentiating into chondrocytes or promoting the proliferation and differentiation of the remaining healthy chondroprogenitors into mature chondrocytes, or both. By releasing trophic factors and cell-to-cell interactions, MSCs may enhance cartilage regeneration and reduce synovial inflammation in the osteoarthritic joint.

Besides having excellent properties for regeneration of tissues, the immunomodulatory properties of MSCs is also one of their superior characteristics. This makes MSCs as a promising cell source to repair the damage of cartilage tissue and at the same time provide immunomodulatory effect to reduce inflammation in OA. MSCs have been extensively studied for their roles in inflammation. MSCs response to inflammation by homing to the damaged tissues, regulating immune and inflammatory responses at the inflamed areas, thus facilitating repair of the damaged tissues (Figure 3). 

In general, MSCs have the capacity to modulate both innate and adaptive immune responses. MSCs have been reported to modulate cytokine production by the dendritic cell and Th1/Th2 cells [138], block maturation and activation of antigen presenting cell (APC) [139], as well as regulate the production of CD4^+^CD25^+^regulatory cells [140]. MSCs are also prominent for their immunosuppressive effects through the inhibition of T-lymphocyte activation and proliferation as well as modulating the expression of pro-inflammatory cytokines and chemokines [141,142]. In addition, immunomodulation by MSCs is reported to be mediated via both direct cell to cell contact and also through secretion of soluble factors such as PGE2, indoleamine 2,3-dioxygenase (IDO) and NO [143]. These aforementioned mechanisms could contribute to resolution of inflammation in OA. However, it is still unclear how MSCs facilitate tissue regeneration and inflammation process. Many studies have shown that MSCs’ paracrine activity may play some role in modifying the milieu of the injured tissue, resulting in more favorable circumstances for tissue regeneration [67]. MSCs secrete cytokines to reduce inflammation in surrounding tissues and initiate cartilage repair, which is followed by chondrogenic proliferation and the secretion of ECM proteases and growth factors such as TGF-β IGF-1 and FGF [144].

Intra-articular administration of MSCs into arthritic shoulder of the rat model indicated downregulation of ADAMTS5 expression in the joint cartilage, but increased expression of TNF-α stimulated gene/protein 6 (TSG-6) and inhibited the expression of anti-calcitonin gene related peptide (CGRP) indicating suppression of the central sensitization of pain [145]. Another study using intra-articular administration of umbilical cord-derived MSCs (UC-MSCs) indicated anti-inflammatory and anti-catabolic effects of UC-MSCs as demonstrated by decreased expression of the pro-inflammatory cytokines and MMPs in the synoviocytes of the rabbit model [146].

Many in vitro and in vivo investigations have shown that MSC-derived EVs have significant anti-inflammatory and regenerative effects in OA models. In an experimental study by Vonk et al., EV isolated from human bone marrow MSC altered the TNF-α-mediated upregulation of COX2 and pro-inflammatory interleukins, i.e., IL-1α, IL-1β, IL-6, IL-8 and IL-17 when co-cultured with TNF-α-stimulated OA chondrocyte [147]. EVs derived from human AD-MSCs demonstrated chondroprotective effects by decreasing the release of inflammatory mediators (e.g., TNF-, IL-6, PGE2 and NO) and MMP activity, while increasing the production of the anti-inflammatory cytokine IL-10. In a recent study, exosomes from embryonic MSCs reduced inflammatory response, enhanced cartilage repair and subchondral bone healing, reversed IL-1-mediated inhibition of sulfated glycosaminoglycan synthesis, and decreased IL-1-induced production of nitric oxide and MMP-13 via adenosine-mediated activation of AKT, ERK and AMPK pathways in an OA model of the temporomandibular joint of immunocompetent rats [54]. The immunomodulatory properties of MSCs and MSC-derived EVs may help decrease inflammation and prevent the progression of OA, making them potential therapeutic sources for OA.

## 8. Conclusions and Future Perspective

OA is the most common joint disorder worldwide especially among the elderly. However, OA remains irreversible as there is no effective treatment to cure it. When OA becomes severe, the only option for therapy, other than pain medication, is joint replacement. With current advancement in implants, although joint replacement is an effective therapy for symptomatic end-stage osteoarthritis, outcomes can be poor. The advancements in cell-based therapy offer interesting approaches for the treatment of OA. ACI has been successfully clinically applied in OA patients, however there are a number of issues and concerns surrounding this therapeutic approach as mentioned above. Due to these issues, MSC-based therapy is a promising therapeutic strategy to overcome the difficulty of treating OA. MSCs have chondrogenic properties and exhibit immunomodulatory activity that can help to minimize inflammation in OA. In this review, we described the pre-clinical and clinical studies using different sources of MSC with various approaches. In addition, other approaches using the MSC-based EV in the treatment of OA and their immunomodulatory effects are also described in this review. Despite the fact that MSCs are showing promising results in OA patients, however there are several factors to consider to further improve the treatment and management of the stem cell isolation. The mechanism of action of the stem cell, as well as its chondrogenic potential and immunomodulatory effects in the OA model, must be thoroughly investigated before a new treatment strategy can be implemented. Proper isolation, delivery and management of the stem cells isolated from the patient must be carefully evaluated to avoid immunological rejection and to make sure optimal number of the MSC is obtained. Recently, MSC-based EV offers promising therapeutic potential and immunomodulatory capacity which warrants further investigation. Furthermore, innovative methods using MSC and biomaterial construct for cartilage tissue engineering must be investigated to improve the chondrogenic potential and immunomodulatory properties of the system for OA treatment.

## Data Availability

No new data were created or analyzed in this study. Data sharing is not applicable to this article.
